# Effectiveness of the Minder Mobile Mental Health and Substance Use Intervention for University Students: Randomized Controlled Trial

**DOI:** 10.2196/54287

**Published:** 2024-03-27

**Authors:** Melissa Vereschagin, Angel Y Wang, Chris G Richardson, Hui Xie, Richard J Munthali, Kristen L Hudec, Calista Leung, Katharine D Wojcik, Lonna Munro, Priyanka Halli, Ronald C Kessler, Daniel V Vigo

**Affiliations:** 1 Department of Psychiatry Faculty of Medicine University of British Columbia Vancouver, BC Canada; 2 School of Population and Public Health Faculty of Medicine University of British Columbia Vancouver, BC Canada; 3 Faculty of Health Sciences Simon Fraser University Burnaby, BC Canada; 4 Menninger Department of Psychiatry & Behavioural Sciences Baylor College of Medicine Houston, TX United States; 5 Department of Health Care Policy Harvard Medical School Boston, MA United States

**Keywords:** mental health, substance use, college students, mobile interventions, digital interventions, randomized controlled trial, mobile phone

## Abstract

**Background:**

University attendance represents a transition period for students that often coincides with the emergence of mental health and substance use challenges. Digital interventions have been identified as a promising means of supporting students due to their scalability, adaptability, and acceptability. *Minder* is a mental health and substance use mobile app that was codeveloped with university students.

**Objective:**

This study aims to examine the effectiveness of the *Minder* mobile app in improving mental health and substance use outcomes in a general population of university students.

**Methods:**

A 2-arm, parallel-assignment, single-blinded, 30-day randomized controlled trial was used to evaluate *Minder* using intention-to-treat analysis. In total, 1489 participants were recruited and randomly assigned to the intervention (n=743, 49.9%) or waitlist control (n=746, 50.1%) condition. The *Minder* app delivers evidence-based content through an automated chatbot and connects participants with services and university social groups. Participants are also assigned a trained peer coach to support them. The primary outcomes were measured through in-app self-assessments and included changes in general anxiety symptomology, depressive symptomology, and alcohol consumption risk measured using the 7-item General Anxiety Disorder scale, 9-item Patient Health Questionnaire, and US Alcohol Use Disorders Identification Test–Consumption Scale, respectively, from baseline to 30-day follow-up. Secondary outcomes included measures related to changes in the frequency of substance use (cannabis, alcohol, opioids, and nonmedical stimulants) and mental well-being. Generalized linear mixed-effects models were used to examine each outcome.

**Results:**

In total, 79.3% (589/743) of participants in the intervention group and 83% (619/746) of participants in the control group completed the follow-up survey. The intervention group had significantly greater average reductions in anxiety symptoms measured using the 7-item General Anxiety Disorder scale (adjusted group mean difference=−0.85, 95% CI −1.27 to −0.42; *P*<.001; Cohen *d*=−0.17) and depressive symptoms measured using the 9-item Patient Health Questionnaire (adjusted group mean difference=−0.63, 95% CI −1.08 to −0.17; *P*=.007; Cohen *d*=−0.11). A reduction in the US Alcohol Use Disorders Identification Test–Consumption Scale score among intervention participants was also observed, but it was not significant (*P*=.23). Statistically significant differences in favor of the intervention group were found for mental well-being and reductions in the frequency of cannabis use and typical number of drinks consumed. A total of 77.1% (573/743) of participants in the intervention group accessed at least 1 app component during the study period.

**Conclusions:**

In a general population sample of university students, the *Minder* app was effective in reducing symptoms of anxiety and depression, with provisional support for increasing mental well-being and reducing the frequency of cannabis and alcohol use. These findings highlight the potential ability of e-tools focused on prevention and early intervention to be integrated into existing university systems to support students’ needs.

**Trial Registration:**

ClinicalTrials.gov NCT05606601; https://clinicaltrials.gov/ct2/show/NCT05606601

**International Registered Report Identifier (IRRID):**

RR2-10.2196/49364

## Introduction

### Background

University attendance is a transitional period in which many students experience novel stressors related to moving away from home, navigating new social environments, and managing increased educational and financial demands in the absence of their traditional support systems [1,2]. The transition to attending university also coincides with the peak period of onset of many mental disorders, including mood, anxiety, and substance use disorders [3,4]. Studies have documented the high rates of mental health and substance use problems experienced by university students [5], with research also indicating that students with preexisting mental health problems can experience a worsening of their conditions following the transition to attending university [6].

Despite this high need, most students experiencing mental health problems do not receive treatment [7]. Research investigating help seeking among university students indicates that, compared to structural barriers, attitudinal barriers are the most important reasons for not seeking help [8]. The most commonly cited reason students give for not seeking help is a preference for handling things on their own [7,8]. One way of adapting interventions to align with this preference is to provide students with tools that are self-guided and allow them autonomy over how and when to use the tools provided. e-Interventions can be accessed by users at any time and have been demonstrated to be effective in improving various mental health [9] and substance use outcomes among university students [10]. These interventions have also been identified as key components in proposed models of care for universities [11]. While much of the literature on e-interventions has been focused on web-based tools, mobile apps have been identified as a promising means of delivering mental health interventions due to not only the increase in smartphone use but also the wide range of interventions that can be delivered through mobile platforms [12,13].

Given the range of challenges faced by university students, including the high rates of disorder-level and subclinical mental health and substance use problems, transdiagnostic approaches to early intervention and prevention may be beneficial for this population [14]. Developing this type of intervention requires the use of a holistic, student-centered design approach to identify evidence-based condition-specific and cross-cutting opportunities for intervention. Furthermore, the intervention needs to be aligned with the perceived needs and preferences of students to ensure meaningful engagement [15]. On the basis of these requirements, we codeveloped a mental health and substance use mobile app called *Minder* for Canadian university students. This participatory codevelopment process involved significant input from students through the creation of a Student Advisory Committee, usability testing via a virtual boot camp (ie, individual user-testing combined with a web-based survey), focus groups, and a pilot feasibility study [16].

### Objectives

The objective of this study was to test the effectiveness of the *Minder* mobile app in improving mental health and substance use outcomes in a general population of university students.

## Methods

### Trial Design

This study was based on a 2-arm, parallel-assignment, single-blinded (the statistician was blinded), 30-day randomized controlled trial with 1 intervention group and 1 waitlist control group. The study was registered at ClinicalTrials.gov (NCT05606601), and a full study protocol has been published [17]. No significant changes to the trial protocol or intervention content were made during the trial period; however, several minor adjustments, along with a description of minor technical issues, can be found in Multimedia Appendix 1.

### Ethical Considerations

Ethics approval was obtained from the University of British Columbia (UBC) Behavioural Research Ethics Board on January 6, 2022 (ethics ID: H21-03248). Informed consent was obtained through a web-based self-assessment questionnaire at the beginning of the study. Participants were informed of their ability to opt out at any point within the study by emailing the research team. Identifiable data were stored in data files within the app backend, which were separate from all deidentified app use and survey data. This information can only be linked using a unique study ID number. Participants received a CAD $10 (US $7.4) gift card for completion of the baseline survey and an additional CAD $10 (US $7.4) gift card for completion of the 30-day follow-up survey.

### Participants

The study was conducted at the UBC Point Grey (Vancouver, British Columbia, Canada) campus. Participants needed to confirm their eligibility using a web-based self-assessment questionnaire before registering and consenting to the study. The inclusion criteria were as follows: students currently enrolled at the UBC Vancouver campus, aged ≥17 years, having access to and being able to use a smartphone with Wi-Fi or cellular data, and speaking English. The only exclusion criterion was based on a single screening question assessing suicidality risk (“We want to make sure that this app is appropriate for you at this time. Do you have a current suicidal plan [i.e., a plan to end your life]?”). Anyone endorsing a current suicidal plan (ie, answering “yes”) was prevented from registering and was instead provided with a list of local crisis resources. The eligibility criterion of being a current UBC student was confirmed using a unique student log-in checkpoint as part of the registration process. This process also ensured that each student could only enroll once.

### Recruitment and Consent

Given the large sample size needed for this study, many different recruitment methods were used. Online recruitment occurred through various social media platforms and a linked ongoing Student E-Mental Health trend study [18]. Recruitment also occurred through in-person and on-campus engagements, such as setting up informational booths at the university, displaying posters about the study, visiting in-person and online classes, having professors share study information with their classes, and contacting student groups to share information with their members. Paid bus and bus stop advertisements at the university were also used. A more detailed description of the recruitment methods can be found in the study protocol for this trial [17].

Participants’ consent was obtained using Qualtrics (Qualtrics International Inc), a web-based form, after completing the eligibility screening. The consent form indicated that participants would either gain access to the full app immediately or in 30 days following completion of the final survey. Individual accounts were created for each participant and sent to them with a link to download the app. Upon downloading the app and completing the baseline survey, participants were randomly assigned through the app to the intervention group, which received full access to the *Minder* app, or to the control group, which only had access to a restricted version of the app that included a short introduction video and the baseline and follow-up surveys. Participants received a CAD $10 (US $7.40) gift card for completion of the baseline survey and an additional CAD $10 (US $7.40) gift card for completion of the 30-day follow-up survey; however, the use of the app itself was not remunerated.

### Randomization and Intervention

Participants were randomized using a custom-developed automated process incorporated directly into the mobile app following completion of the baseline survey. The system assigned participants to either the intervention or control group using a predetermined block randomization list (1:1 randomization in blocks of 10) stratified for past drug use (any lifetime use of opioids or nonmedical stimulants). Stratification by past drug use was used to account for the low number of students using these substances and the need to ensure that they were evenly distributed across the study groups. The randomization lists (1 for each stratification group) were generated using the web-based stratified block randomization list creator in the clinical trial software Sealed Envelope (Sealed Envelope Ltd) [19]. The intervention and control groups completed the main assessments of the primary and secondary outcomes at baseline and the 30-day follow-up. The intervention group was also prompted to complete a short survey at 2 weeks that consisted of a limited set of questions on anxiety and depression symptoms.

The *Minder* mobile app was codeveloped with university students and professionals with the goal of creating an engaging self-directed tool for students to improve their mental health and manage substance use. The intervention is designed for a general population of students and, thus, addresses a wide range of challenges related to postsecondary student life, including managing emotions, relationships, well-being, and university life. The self-directed nature of the app also allows students to access features when needed. The codevelopment process consisted of ongoing student input through student staff members and volunteers along with several phases of purposeful student engagement and feedback. Further details on the codevelopment process can be found in the study by Vereschagin et al [16].

**Figure 1:**
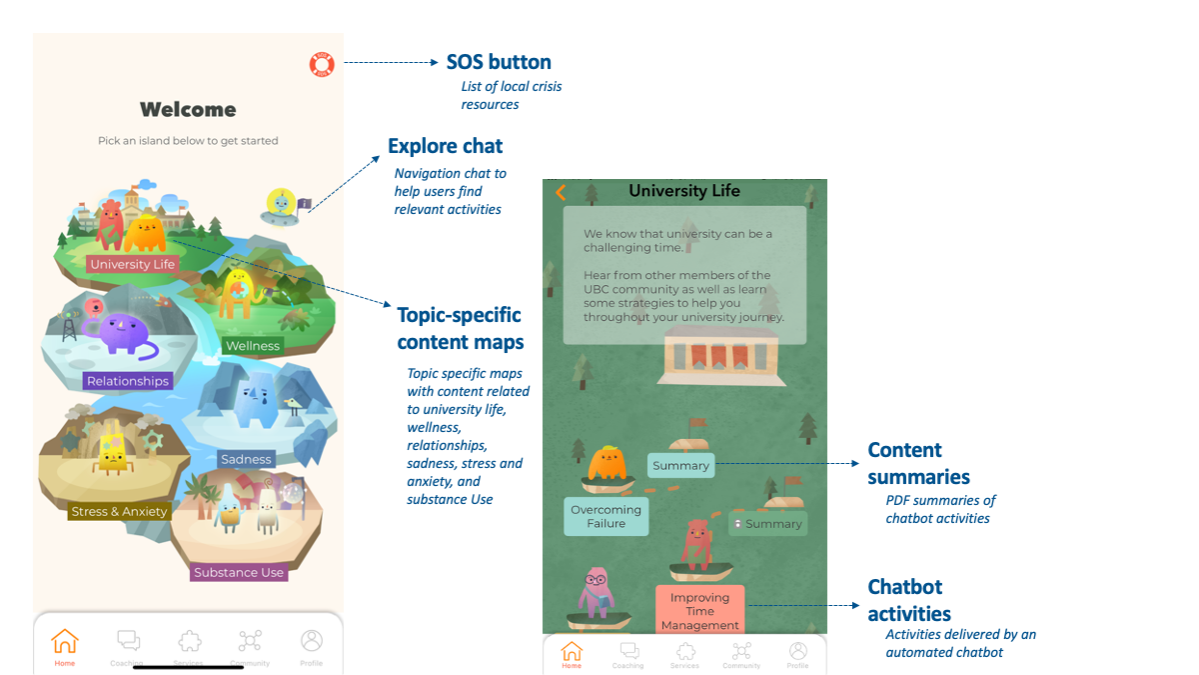
Minder home screen and content maps. The home screen contains 6 topic islands that users can select from. Each island leads to a separate map with chatbot activities and summaries. Additional features include the SOS button and the Explore Chat that guides users to activities.

Participants who were randomized to the intervention group were given full access to the *Minder* app and instructed to use it as they wanted. They were also presented with a tutorial video outlining the different features of the app. The *Minder* intervention consists of 4 main components: Chatbot Activities, Services, Community, and Peer Coaching. The chatbot activities consist of an automated preprogrammed chatbot that delivers evidence-based messages and videos. The content is based primarily on cognitive behavioral therapy and psychoeducation; however, there is also content adapted from dialectical behavioral therapy, mindfulness, metacognitive training, and motivational interviewing. The content sections are presented on a home page map with several islands: *University Life*, *Wellbeing*, *Relationships*, *Sadness*, *Stress & Anxiety*, and *Substance Use* (Figure 1). There is also an *Explore Chat* located on the home page map that guides participants to select an activity that may be relevant to their current needs. A full list of the content included can be found in Multimedia Appendix 2 [16]. Most of the chat activities also contain a summary page that is unlocked upon completion of the chat activity and allows participants to review content they learned at a later time. In addition, several chat activities contain specific practice components that also become unlocked after the main activity is complete.

**Figure 2:**
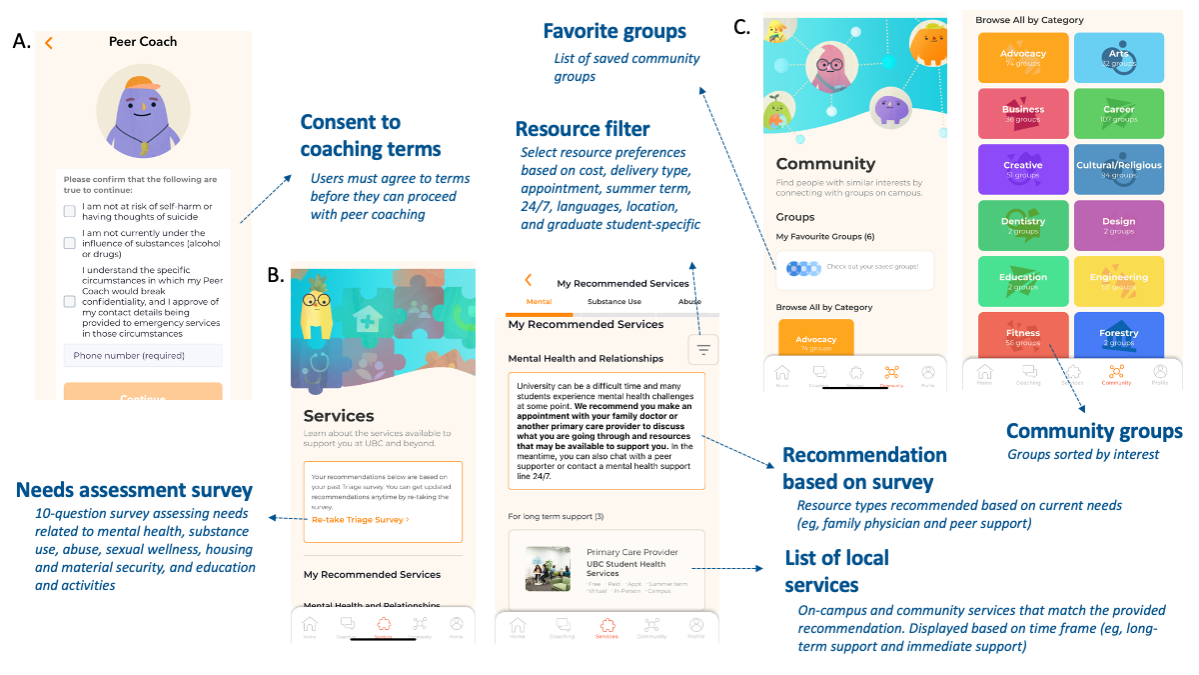
Peer coach, services, and community components. (A) The peer coach component allows users to connect with trained student volunteers, (B) the services component matches users with resources, and (C) the community component lists student groups by interest.

The Peer Coaching component consists of trained volunteers assigned to each participant. These peer coaches reach out to participants at the beginning of the trial and midway through. They can provide support in navigating the app or nonclinical peer support based on active listening and problem-solving. Peer coaches can communicate with participants through an in-app chat asynchronously or synchronously through scheduled appointments delivered over in-app chat message or audio or video call. Before engaging with peer coaches, participants must provide a phone number that can be used to contact them in a crisis situation and affirm that they are not currently at risk of self-harm or having suicidal thoughts, are not under the influence of substances, and understand the circumstances in which confidentiality would need to be broken (ie, crisis situations or abuse of a minor; Figure 2A).

The Services component consists of a 10-question survey tool that provides participants with recommendations for resources based on their current needs and preferences (Figure 2B). The survey tool and recommendations were adapted from a previously developed tool for university students [20] and can be completed multiple times to receive new recommendations. Recommendations are provided for 6 areas related to student well-being: mental health and relationships, substance use, abuse, sexual wellness, housing, and education and activities. An additional safety component was added so that participants who based on the services survey were considered to be at high risk of suicidality (ie, plan for suicide or recent attempt and thoughts of hurting others) were asked to consent to provide their contact information and receive an expedited appointment with the university counseling services.

The Community component consists of a searchable directory of student groups or clubs at the university that are sorted by interest (eg, volunteering, arts, and advocacy; Figure 2C).

The *Minder* app also contains several other general features. An SOS button appears in the top corner of the home page and provides a list of crisis resources if needed (Figure 1). The settings page allows participants to update their username and password as well as change their avatar. Several types of push notifications were delivered through the app. General notifications were sent on days 4, 18, and 24. Reminders to complete the 2-week and 30-day follow-up surveys were sent as push notifications and via automated email reminders on those dates. Additional email reminders were sent on days 35 and 41 to remind participants to complete the follow-up survey if they had not done so already.

Participants who were randomized to the control group had access to a locked version of the app that only allowed them to complete the baseline survey and view a short introduction video that appears before the log-in screen. Following completion of the baseline survey, they received a pop-up message telling them that they would be notified when it was time to complete the next survey. The app was then locked so that control participants were not able to access any other areas of the app. At 30 days, participants were notified that it was time to complete the 30-day follow-up survey within the app, and this survey became unlocked. The app provided push notifications and automated email reminders to complete the follow-up survey on day 30 as well as additional email reminders on days 35 and 41 if the survey had not yet been completed.

Individuals who consented to participate in the study and received an invitation email to download the app but did not complete the baseline survey received additional email reminders at 7 days, at approximately 20 days, and several months later following the creation of their account.

The participants’ use of the app components was recorded through the app back-end system. This included starting each of the chatbot activities, completing the services survey and receiving recommendations, viewing community groups, and communicating with a peer coach.

### Measures

The main assessments of the primary and secondary outcomes were collected through self-assessment within the Minder app at baseline and at the 30-day follow-up. The 30-day follow-up survey had to be completed within 44 days of the beginning of the baseline survey (30 days plus 2 weeks to accommodate the use of reminders) for the participants to be included in the analysis.

#### Primary Outcomes

The primary outcomes assessed in this study were changes in general anxiety symptomology, depressive symptomology, and alcohol consumption risk from baseline to follow-up at 30 days. All outcomes were assessed using self-report questionnaires completed directly in the mobile app.

Anxiety symptoms were assessed using the 7-item General Anxiety Disorder scale (GAD-7) assessment—a commonly used self-report scale that assesses symptoms of generalized anxiety [21]. Each GAD-7 question is scored from 0 (*not at all*) to 3 (*nearly every day*), with total scores ranging from 0 to 21 and higher scores indicating a worse outcome (ie, greater frequency of anxiety symptoms).

Depressive symptoms were assessed using the 9-item Patient Health Questionnaire (PHQ-9) self-report scale [22]. Each of the 9 questions is scored from 0 (*not at all*) to 3 (*nearly every day*). The total scores range from 0 to 29, with higher scores indicating a worse outcome (ie, a greater frequency of depressive symptoms).

Alcohol consumption risk was assessed using the US Alcohol Use Disorders Identification Test–Consumption Scale (USAUDIT-C) [23]. The USAUDIT-C is a 3-item self-report scale adapted from the consumption questions in the Alcohol Use Disorders Identification Test (AUDIT) [24]. Compared to the AUDIT, the USAUDIT-C includes expanded response options for the first 3 AUDIT questions—from 5 to 7 categories—to allow for more precise measurements when accounting for differences in standard drink sizes and cutoff limits. The higher the total score on the USAUDIT-C, the greater the respondent’s alcohol consumption and related risk [23].

#### Secondary Outcomes

A range of secondary outcomes, including reduced use of other substances and additional mental health constructs that the *Minder* app was theorized to affect, were also assessed. For the purposes of this study, we focused on examining changes from baseline to follow-up at 30 days in frequency of substance use (cannabis, alcohol, opioids, and nonmedical stimulants) and mental well-being.

Frequency of cannabis use was assessed using a single self-report question on frequency of cannabis consumption in the previous 30 days. The 3 questions on the USAUDIT-C assessed unique dimensions of alcohol consumption. Frequency of alcohol use was assessed using responses to the first question on the USAUDIT-C, which asks how often participants have a drink containing alcohol. The number of drinks consumed in a typical drinking session was assessed using the second question on the USAUDIT-C, which asks participants how many drinks containing alcohol they have on a typical day when drinking. Frequency of binge drinking was assessed using the third question in the USAUDIT-C, which asks participants how often they have ≥5 (if sex at birth was male) *or* ≥4 (if sex at birth was female) drinks on 1 occasion.

Frequency of any opioid use in the previous 30 days was assessed using self-reported questions that asked about any pharmaceutical opioid (eg, oxycodone; morphine; hydromorphone; meperidine; fentanyl patches; and codeine or codeine-containing products such as Tylenol 1, 2, or 3) with a physician’s prescription and taken as prescribed; any pharmaceutical opioid (eg, oxycodone; morphine; hydromorphone; meperidine; fentanyl patches; and codeine or codeine-containing products such as Tylenol 1, 2, or 3) either without a physician’s prescription or in larger doses than prescribed to get high, buzzed, or numbed out; and any street opioid (eg, heroin and fentanyl) or any other opioid obtained “on the street.” The final opioid use outcome was defined as the most frequent number among any prescribed, nonprescribed, and street opioids.

Frequency of nonmedical stimulant use in the previous 30 days was assessed using self-report questions that asked about the frequency of using any street stimulant (eg, cocaine, crack, methamphetamines, and crystal meth) or prescription stimulant (eg, amphetamine, methylphenidate, and modafinil) either without a physician’s prescription or in larger doses than prescribed to get high, buzzed, numbed out, or help them study or for any other reason. The final nonmedical stimulant use outcome was defined as the most frequent number among any prescription stimulant without a prescription or not as prescribed and any street stimulant. The exact wording and response options for the substance use secondary outcome assessments can be found in Multimedia Appendix 3.

Mental well-being was assessed using the Short Warwick-Edinburgh Mental Wellbeing Scale. The Short Warwick-Edinburgh Mental Wellbeing Scale is a 7-item scale that has been widely validated [25], with total scores ranging from 7 to 35 and higher scores indicating a better outcome (ie, higher positive mental well-being).

### Sample Size Estimation

The a priori sample size calculation for a small effect assessed using the PHQ-9, GAD-7, and USAUDIT-C was performed using an effect size defined by Cohen *d*=Δ/σ, where Δ is the group mean difference at the completion of the study and σ is the (pooled) within-group SD [17]. For a small effect size (Cohen *d*=0.2), the sample size required to have 80% power at a *P*=.02 level of significance (ie, 0.05/3 primary outcomes) was 524 in each group. After incorporating a 30% attrition rate, we anticipated requiring 748 participants in each group for a total of 1496 participants for the trial to be adequately powered.

### Statistical Analysis

The study used a single-blinded approach in which only the statistician, who was external to the team, was blinded to the treatment group assignment when examining the primary hypotheses. The primary analysis was intention-to-treat (ITT) including all participants who completed the baseline assessment and were randomized to either the control or intervention group following the analysis plan prespecified in our protocol paper [17]. The analysis considered the following 2 features of the trial design: 3 primary end points and the use of block randomization.

For the 3 primary end points (GAD-7, PHQ-9, and USAUDIT-C scores), a global test for the null hypothesis of no treatment difference in all primary end points between the control and treatment groups was conducted first using a multivariate analysis of covariance for correlated data on GAD-7, PHQ-9, and USAUDIT-C scores at 30 days after the baseline, adjusting for their values at baseline and randomization blocks. Compared with testing each outcome separately, the advantages of the global test from joint modeling included more parsimonious hypothesis tests and mitigated concerns related to multiple testing [26-28] as well as pooling of the information over the correlated outcomes to increase study power, especially with missing outcomes [29]. If the global test rejected the null hypothesis and we concluded that there was an intervention effect on at least 1 of the 3 end points, we would then analyze each end point separately to identify which of the 3 study end points were affected by the intervention. We then used the sequential Hochberg correction method [30] to control the overall familywise error rate at α=.05 when testing the hypothesis for each individual primary end point. For baseline characteristics, 2-sample *t* tests (2-tailed) were used to compare means, chi-square tests were used to compare proportions, and the Kruskal-Wallis test was used to test for differences in medians.

The general approach for analyzing all the types of individual study outcomes separately (including the 3 primary end points and the secondary end points) was generalized linear mixed-effects models (GLMMs) for clustered measures with the randomization block as the clustering variable. These models can handle a wide range of outcome types, including continuous, binary, ordinal, and count, and can account for the correlations among observations within the same cluster. GLMMs have been widely used for conducting ITT analysis in randomized controlled trials with missing outcome data and can account for data missing at random (MAR) without the need to model why data are missing or to perform explicit imputations of the missing values [31]. Specifically, we analyzed each primary end point using a linear mixed-effects model, a special case of the GLMM.

For secondary outcomes, we used linear mixed-effects models to analyze mental well-being measures and a mixed-effects quasi–Poisson regression model with a log link (a special case of GLMM) for substance use frequency measures, and the zero-inflated Poisson was used for the number of drinks to deal with the excess zero counts. The treatment effect on an outcome at 30 days was assessed in these models with the treatment allocation as the main explanatory variable and with adjustment for baseline outcome assessment value as a fixed effect and the randomization block as a random effect. Robust empirical sandwich SE estimates that are robust to model misspecifications (eg, nonnormal error terms) were used for statistical inference.

The results from these GLMMs for the analysis are reported in the Results section as the model-adjusted differences in the group mean values of the continuous end points between the intervention and control groups. These intervention effect estimates, 95% CIs, and *P* values were obtained from the aforementioned GLMMs, clustering on randomization block effects and adjusting for the baseline outcome values. Standardized effect sizes were calculated for continuous scores by dividing the adjusted mean differences by the SDs across all participants at baseline. The incidence rate ratios (IRRs) from Poisson regression through GLMM and zero inflation for the number of drinks are also reported.

To evaluate the robustness of the results to alternative assumptions regarding missing data, sensitivity analyses were conducted on the primary outcomes via (1) using selection models to measure the potential impacts of data missing not at random [32,33] and (2) adjusting analysis for additional baseline covariates potentially predictive of missing data. All the primary analyses were conducted in SAS (version 9.4; SAS Institute), except for the sensitivity analysis of the selection models, which was conducted in the *isni* package in R (version 3.4; R Foundation for Statistical Computing) [34], whereas secondary analyses were conducted in Stata (version 15.1; StataCorp) [35].

### Data and Privacy

Many steps were taken to ensure the privacy of participants. Each participant received an individual account that had a unique username and password. They were then able to change this password upon logging in. Only participant emails and phone numbers (if provided through access to peer coaching) were stored within the app; names were used only for consent and were never entered into the app. Instead, participants could create a unique username within the app that they were informed should not include their full name. Identifiable data (email and phone number) were stored in data files within the app back end separate from all the app use and survey data, which were recorded with only a study ID number.

## Results

### Recruitment

Recruitment initially began on September 4, 2022, during an on-campus student orientation event where interested students were asked to provide contact information to receive a follow-up email; however, participants were not provided with app download information and user accounts to begin the study until September 28, 2022, due to technical delays. Recruitment concluded on June 2, 2023, with the last participant beginning the trial on June 11, 2023.

### Participant Flow

**Figure 3:**
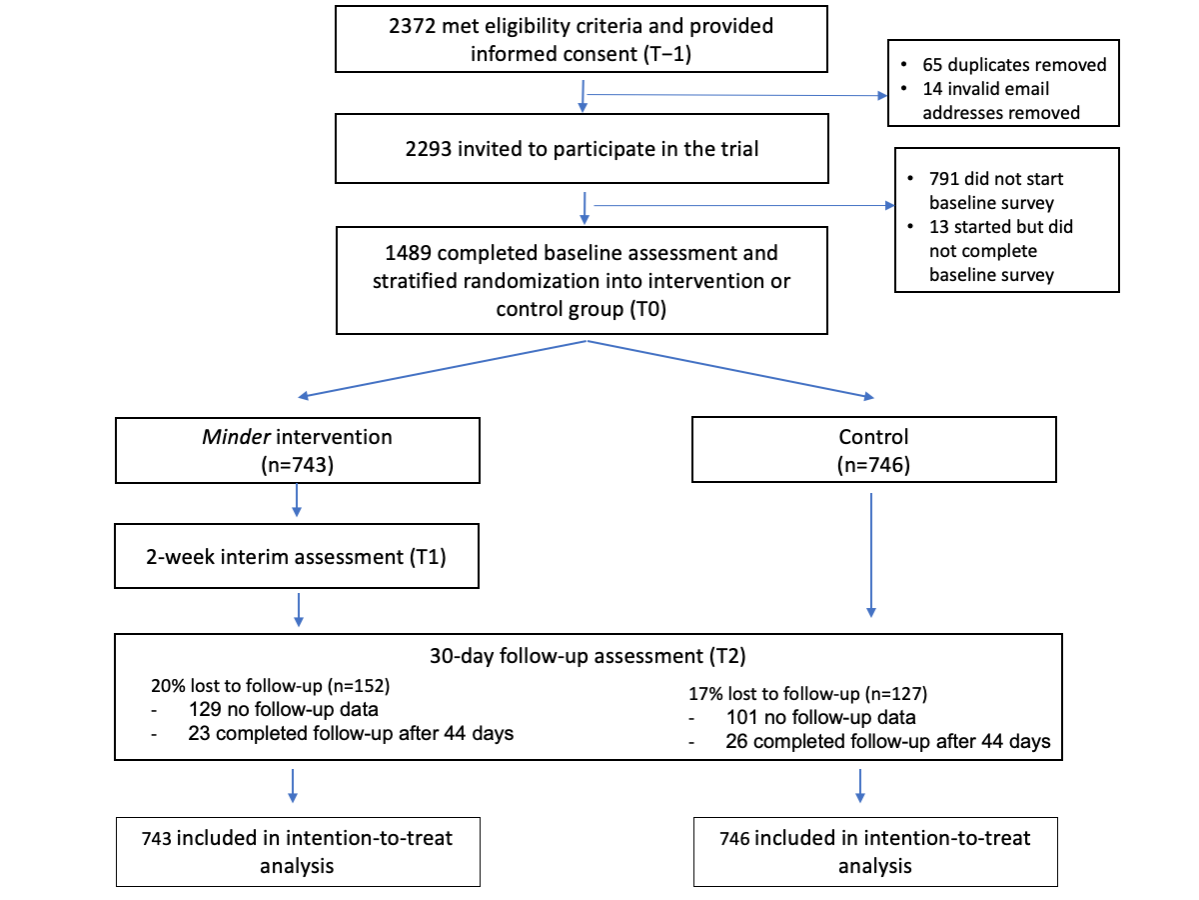
CONSORT (Consolidated Standards of Reporting Trials) flow diagram completed with participant information.

In total, 2293 individuals were invited to participate in the trial following eligibility screening and provision of informed consent. Of those 2293 individuals, 1489 (64.9%) participants completed the baseline survey and were randomized into the trial, with 743 (49.9%) of the 1489 participants in the intervention group and 746 (50.1%) in the control group. A total of 79.3% (589/743) of participants in the intervention group and 83% (619/746) of participants in the control group completed the 30-day follow-up survey within the specified 44-day period to be included in the analysis (ie, 279/1489, 18.7% of participants who completed the baseline survey did not complete the 30-day follow-up survey within 44 days and were therefore considered lost to follow-up). Additional information on the participant flow is shown in Figure 3.

### Participant Characteristics

The baseline characteristics of the participants in the intervention and control groups are presented in Table 1. The median age of the participants was 20 years, and 70.3% (1045/1487) self-identified as women. In terms of mental health, 33.8% (455/1347) reported a history of anxiety, and 38.9% (576/1481) reported moderate or greater levels of recent anxiety (ie, total score of ≥10) based on the GAD-7. A history of depression was reported by 28.4% (382/1347) of participants, with 43.8% (645/1474) reporting moderate or greater levels of recent depressive symptomology (ie, total score of ≥10) on the PHQ-9. The intervention group had higher baseline scores on both the GAD-7 (*P*=.02) and PHQ-9 (*P*=.02)*.* No other statistically significant differences in baseline characteristics were found between the intervention and control groups (Table 1).

**Table 1:** Participant characteristics at baseline (N=1489)^a^.

Characteristics				Overall		Intervention (n=743)		Control (n=746)		*P* value	
Age (y; n=1042), median (IQR)				20 (19-23)		20 (19-23)		20 (19-23)		.54	
**Student year (n=1487), n (%)**											.28
	Undergraduate: year 1			384 (25.8)		195 (26.3)		189 (25.3)			
	Undergraduate: year 2			280 (18.8)		126 (17)		154 (20.6)			
	Undergraduate: year 3			264 (17.8)		136 (18.4)		128 (17.2)			
	Undergraduate: year 4 or 5			287 (19.3)		136 (18.4)		151 (20.2)			
	Graduate			206 (13.9)		114 (15.4)		92 (12.3)			
	Other			66 (4.4)		34 (4.6)		32 (4.3)			
**Gender (n=1487), n (%)**											.71
	Woman			1045 (70.3)		527 (71.1)		518 (69.4)			
	Man			364 (24.5)		178 (24)		186 (24.9)			
	Nonbinary			78 (5.2)		36 (4.9)		42 (5.6)			
**Race or ethnicity (n=1454), n (%)**											.66
	Black			18 (1.2)		7 (1)		11 (1.5)			
	East or Southeast Asian			479 (32.9)		240 (33.1)		239 (32.8)			
	Hispanic or Latino			46 (3.2)		24 (3.3)		22 (3)			
	Indigenous or First Nations			28 (1.9)		14 (1.9)		14 (1.9)			
	South Asian			198 (13.6)		102 (14)		96 (13.2)			
	West or Central Asian			58 (4)		30 (4.1)		28 (3.8)			
	White			436 (30)		210 (28.9)		226 (31)			
	Multiracial			187 (12.9)		95 (13.1)		92 (12.6)			
	Other			4 (0.3)		4 (0.6)		0 (0)			
**Mental health diagnosis or treatment history (n=1347), n (%)**											
	History of anxiety (yes)			455 (33.8)		233 (34.5)		222 (33)		.57	
	History of depression (yes)			382 (28.4)		196 (29)		186 (27.7)		.58	
	History of substance (alcohol or drug) use (yes)			19 (1.4)		9 (1.3)		10 (1.5)		.81	
	History of other mental health disorders (yes)			353 (26.2)		178 (26.4)		175 (26)		.89	
**Current mental health and substance use**											
	**GAD-7^b^ (n=1481), mean (SD)**			8.7 (5)		9.0 (5)		8.4 (5)		.02	
		GAD-7≥10 (moderate and above), n (%)	576 (38.9)		297 (40.1)		279 (37.7)		.35		
	**PHQ-9 ^c^ n=1474), mean (SD)**			9.2 (5.8)		9.6 (5.9)		8.9 (5.6)		.02	
		PHQ-9≥10 (moderate and above), n (%)	645 (43.8)		326 (44.5)		319 (43)		.55		
	USAUDIT-C^d^ (n=1483), mean (SD)			3.5 (3.2)		3.6 (3.2)		3.5 (3.3)		.31	
	SWEMWS^e^ (n=1474), mean (SD)			21.5 (4.2)		21.3 (4.3)		21.6 (4.1)		.19	

^a^Variations in the total n values for each characteristic were due to missing responses. We used 2-sample *t* tests (2-tailed) to compare means, chi-square tests to compare proportions, and the Kruskal-Wallis test to test for differences in medians.

^b^GAD-7: 7-item General Anxiety Disorder scale.

^c^PHQ-9: 9-item Patient Health Questionnaire.

^d^USAUDIT-C: US Alcohol Use Disorders Identification Test–Consumption Scale.

^e^SWEMWS: Short Warwick-Edinburgh Mental Wellbeing Scale.

### App Use

Among those in the intervention group (743/1489, 49.9%), 77.1% (573/743) accessed at least 1 app component during the 30-day study period. More specifically, 73.8% (548/743) engaged in 1 or more chatbot activities, 21.8% (162/743) accessed the community component, 27.9% (207/743) completed the services survey, and 17.2% (128/743) accessed a peer coach.

### Outcomes

#### Primary Outcomes

For the 3 primary end points (GAD-7, PHQ-9, and USAUDIT-C scores), a global Wald test with the null hypothesis of no treatment difference in all primary end points between the control and treatment groups was conducted using a marginal multivariate analysis of covariance model for correlated data. This multivariate test of overall group differences was statistically significant (Hotelling test=0.02; *P*=.001; Table 2), indicating that there were group differences in at least 1 of the primary outcomes. We then tested each of the primary outcomes using a GLMM for clustered measures, adjusting for baseline values with the randomization block as the clustering variable, and applied a sequential Hochberg correction method [30] to control the overall familywise error rate at .05 when testing the hypothesis for each individual primary end point. The results of these GLMMs indicated that participants in the intervention group had significantly greater reductions in anxiety (adjusted group mean difference=−0.85, 95% CI −1.27 to −0.42; *P*<.001; Cohen *d*=−0.17, 95% CI −0.26 to −0.09) and depressive (adjusted group mean difference=−0.63, 95% CI −1.08 to −0.17; *P*=.007; Cohen *d*=−0.11, 95% CI −0.19 to −0.03) symptoms than those in the control group (Table 2). Although participants in the intervention group also demonstrated a greater reduction in alcohol risk scores on the USAUDIT-C, this difference was not statistically significant (adjusted group mean difference=−0.13, 95% CI −0.34 to 0.08; *P*=.23).

We conducted the following analysis to quantify the robustness of primary findings to the assumption of data MAR. First, baseline covariates potentially predictive of missing data were added to the GLMM outcome models, and the intervention effect estimates remained similar and yielded qualitatively similar *P* values (Table 2, last 2 columns). Second, selection models were used that permit the missingness probability to depend on the unobserved outcome values after conditioning on the observed data, after which we computed the index of local sensitivity to nonignorability [34]. The index of local sensitivity to nonignorability analysis results are reported in Table 3. The *ISNI/SD* (ISNI divided by SD) column in Table 3 estimates the change in intervention effect estimates for a moderate size of nonrandom missingness, where a 1-SD (SD of the outcome) increase in the outcome is associated with an e^1^=2.7–fold increase in the odds of being observed conditioned on the same values of the observed predictors for missingness. For such moderately sized nonrandom missingness, the changes in the intervention effect estimates were small for both GAD-7 and PHQ-9 scores (Table 3). The *MinNI* column in Table 3 computes the minimum magnitude of nonignorable missingness needed for substantial sensitivity such that the selection bias due to data missing not at random is of the same size as the SE. The smaller the value of the minimum nonignorable missingness, the greater the sensitivity. A minimum nonignorable missingness of 1 is suggested as the cutoff value for important sensitivity [32]. The minimum nonignorable missingness values for both the GAD-7 and PHQ-9 far exceeded 1, indicating that no sensitivity to potential missingness not at random was present for the primary findings. Table 3 shows that both the control and intervention groups had moderate and comparable missing data percentages for both the GAD-7 and PHQ-9, which can explain why our analysis results were insensitive to the MAR assumption.

**Table 2:** Results for the analysis of primary outcomes at baseline and at the 30-day follow-up.

Primary outcome and period			Unadjusted, mean (SD)					Adjusted difference^a^ (primary analysis; 95% CI)			*P* value^b^			Adjusted difference^c^ (robustness checking; 95% CI)			*P* value^d^
			Intervention (n=743)		Control (n=746)												
**GAD-7^e^**																	
	Baseline	9.0 (5.0)^f^		8.4 (5.0)^g^		N/A^h^			N/A			N/A			N/A		
	30 days	7.7 (5.1)^i^		8.2 (5.1)^j^		−0.85 (−1.27 to −0.42)			<.001			−0.82 (−1.25 to −0.39)			<.001		
	Effect size (Cohen *d*)	N/A		N/A		−0.17 (−0.26 to −0.09)			<.001			−0.16 (−0.25 to −0.08)			<.001		
**PHQ-9^k^**																	
	Baseline	9.6 (5.9)^l^		8.9 (5.6)^m^		N/A			N/A			N/A			N/A		
	30 days	8.6 (6.2)^n^		8.8 (5.8)^o^		−0.63 (−1.08 to −0.17)			.007			−0.58 (−1.04 to −0.11)			.01		
	Effect size (Cohen *d*)	N/A		N/A		−0.11 (−0.19 to −0.03)			.007			−0.10 (−0.18 to −0.02)			.01		
**USAUDIT-C^p^**																	
	Baseline	3.6 (3.2)^g^		3.4 (3.3)^q^		N/A			N/A			N/A			N/A		
	30 days	3.1 (3.0)^r^		3.1 (3.1)^s^		−0.13 (−0.34 to 0.08)			.23			−0.12 (−0.32 to 0.09)			.28		

^a^Adjusting for block numbers and baseline outcome values.

^b^Global test: *P*=.001.

^c^Adjusting for age, gender, student status, race, substance use at baseline, block numbers, and baseline outcome values.

^d^Global test: *P*=.003.

^e^GAD-7: 7-item General Anxiety Disorder scale.

^f^n=741.

^g^n=740.

^h^N/A: not applicable.

^i^n=575.

^j^n=589.

^k^PHQ-9: 9-item Patient Health Questionnaire.

^l^n=732.

^m^n=742.

^n^n=563.

^o^n=587.

^p^USAUDIT-C: US Alcohol Use Disorders Identification Test–Consumption Scale.

^q^n=743.

^r^n=559.

^s^n=590.

**Table 3:** Robustness checking of group comparison of primary end points to the assumption of data missing at random^a^.

Primary end points	Missing data at 30 days, n_m_ (%)			ISNI^b^/SD		MinNI^c^
	Intervention (n=743)	Control (n=746)				
GAD-7^d^	168 (22.6)	157 (21.0)	−0.0004		57.7	
PHQ-9^e^	180 (24.2)	159 (21.3)	0.0020		10.3	
USAUDIT-C^f^	184 (24.8)	156 (20.9)	0.0125		1.2	

^a^In the missing data percentage (n_m_/n), n_m_ is the number of participants not completing the outcome assessment at 30 days, and n is the number of participants in the intention-to-treat sample.

^b^ISNI: index of sensitivity to nonignorability; SD refers to the SD of the outcomes.

^c^MinNI: minimum magnitude of nonignorable missingness. A MinNI of <1 indicates sensitivity to data missing not at random, and a MinNI of >1 indicates no sensitivity to data missing not at random.

^d^GAD-7: 7-item General Anxiety Disorder scale.

^e^PHQ-9: 9-item Patient Health Questionnaire.

^f^USAUDIT-C: US Alcohol Use Disorders Identification Test–Consumption Scale.

#### Secondary Outcomes

The GLMM for clustered measures with the randomization block as the clustering variable was also used to test for differences between the intervention and control groups on secondary outcomes without any adjustment for multiple testing. The results of these GLMMs indicated that (Table 4), compared to those in the control group, participants in the intervention group had significantly greater improvements in mental well-being (adjusted mean difference=0.73, 95% CI 0.35-1.11; *P*<.001; Cohen *d*=0.17, 95% CI 0.08-0.26) and were significantly associated with a 20% reduction in their frequency of cannabis use (IRR=0.80, 95% CI 0.66-0.96; *P*=.02) and a 13% reduction in the typical number of drinks consumed when drinking (IRR=0.87, 95% CI 0.77-0.98; *P*=.03). No significant differences were found in frequency of binge drinking (IRR=0.98, 95% CI 0.86-1.13; *P*=.83), frequency of drinking (IRR=0.97, 95% CI 0.88-1.06; *P*=.48), or frequency of any opioid use (IRR=0.62, 95% CI 0.16-2.31; *P*=.48). The impact of the intervention on nonmedical stimulant use could not be assessed due to the small number of nonmedical stimulant users at baseline and follow-up.

**Table 4:** Results for the analysis of secondary outcomes at baseline and at the 30-day follow-up.

Secondary outcome and period		Intervention (n=743)	Control (n=746)	IRR^a^ (95% CI)	*P* value
**Warwick well-being^b^, mean (SD)**					
	Baseline	21.3 (4.3)^c^	21.6 (4.1)^c^	N/A^d^	N/A
	30 days	22.3 (4.5)^e^	21.8 (4.3)^f^	0.73 (0.35-1.11)	<.001
	Effect size (Cohen *d*)	N/A	N/A	0.17 (0.08-0.26)	N/A
**Cannabis use frequency, median category (IQR)**					
	Baseline	0 (0-2^g^)^h^	0 (0-2^g^)^c^	N/A	N/A
	30 days	0 (0-2^g^)^i^	0 (0-2^g^)^j^	0.80 (0.66-0.96)	.02
**Binge drinking frequency, median category (IQR)**					
	Baseline	1 (0-1^g^)^k^	1 (0-1^g^)^l^	N/A	N/A
	30 days	1 (0-1^g^)^m^	1 (0-1^g^)^n^	0.98 (0.86-1.13)	.83
**Drinking frequency, median category (IQR)**					
	Baseline	2 (0-3^g^)^h^	1 (0-2^g^)^o^	N/A	N/A
	30 days	1 (0-3^g^)^p^	1 (0-2^g^)^q^	0.97 (0.88-1.06)	.48
**Number of alcoholic drinks, median category (IQR)**					
	Baseline	1 (0-2^g^)^h^	1 (0-2^g^)^r^	N/A	N/A
	30 days	0 (0-2^g^)^s^	0 (0-2^g^)^t^	0.87 (0.77-0.98)	.03
**Stimulant use frequency, median category (IQR)**					
	Baseline	0 (0-0^g^)^u^	0 (0-0^g^)^v^	No analyses	N/A
	30 days	0 (0-0^g^)^w^	0 (0-0^g^)^q^	N/A	N/A
**Opioid use frequency, median category (IQR)**					
	Baseline	0 (0-0^g^)^v^	0 (0-0^g^)^o^	N/A	N/A
	30 days	0 (0-0^g^)^x^	0 (0-0^g^)^y^	0.62 (0.16-2.31)	.48

^a^IRR: incidence rate ratio.

^b^Adjusted difference.

^c^n=737.

^d^N/A: not applicable.

^e^n=567.

^f^n=587.

^g^Refer to Multimedia Appendix 3 for the category definitions for each secondary measure.

^h^n=742.

^i^n=535.

^j^n=577.

^k^n=743.

^l^n=740.

^m^n=581.

^n^n=616.

^o^n=746.

^p^n=611.

^q^n=645.

^r^n=745.

^s^n=594.

^t^n=621.

^u^n=739.

^v^n=741.

^w^n=610.

^x^n=609.

^y^n=643.

## Discussion

### Principal Findings

*Minder* was codeveloped with students to provide them with a set of self-guided tools to manage their mental health and substance use. In this study, we found that participants in the intervention group who had access to the *Minder* app reported significantly greater average reductions in symptoms of anxiety (GAD-7) and depression (PHQ-9) than those in the control group. This finding aligns with the presentation of “stress and anxiety” and “sadness” as key topic areas within the *Minder* app and each topic having its own dedicated content island. Although these findings showed small effects on average in our sample, this may be related to the fact that, at baseline, only 38.9% (576/1481) of the participants had moderate or greater levels of anxiety and 43.8% (645/1474) had moderate or greater levels of depressive symptoms. Many participants in our sample reported only mild or no symptoms of anxiety or depression, which may have contributed to the finding of a small effect on average. Recent reviews on the effects of smartphone apps on anxiety and depression have found larger effects than those reported in this study; however, most studies recruit participants with clinical-level problems [12,13]. A meta-analysis examining internet interventions for university students found small effect sizes for anxiety and depression [36]. The *Minder* intervention group also demonstrated significant improvements in mental well-being in our analysis of secondary outcomes, which reflects a more general positive domain of mental health that may be more relevant to students without existing mental health concerns.

One of the key decisions in this trial was to include students with few or no symptoms of anxiety or depression. Providing interventions to nonclinical populations makes them more accessible to the large proportion of the population who may not meet clinical diagnostic criteria but may still experience occasional mental health challenges that can be addressed using existing tools (eg, via app-based cognitive behavioral therapy) [37]. In addition, this study used an ITT analysis that included all participants who were randomized regardless of whether they used the intervention. There was also no minimum amount of required content for participants to complete, nor were they remunerated for their use of the app. This pragmatic approach provided an approximation of the average effect of *Minder* on mental health and substance use outcomes in a university population if it were to be made available to everyone. As outlined in our study protocol, we plan to complement these findings with additional secondary analyses to examine the impact of *Minder* in subgroups of participants defined by the extent of their app use and baseline mental health and sociodemographic characteristics.

Although we did not find evidence of an effect on overall alcohol consumption risk in our primary outcome analyses of the USAUDIT-C, we did find significant reductions in cannabis use frequency and the typical number of alcoholic drinks consumed in a drinking session in our analysis of secondary outcomes. Reduction in number of drinks consumed when drinking and reducing alcohol-related harms were a main focus of the alcohol intervention content in the *Minder* app. For example, there was an activity in the app that encouraged users to set a goal for how many drinks they would consume in a drinking session and then track the number consumed in real time using the app. In addition, psychoeducational and motivational interviewing content focused on reducing harms associated with drinking, including following lower-risk drinking guidelines or cutting back on alcohol use. Similarly, cannabis use content focused on following lower-risk cannabis use guidelines, such as reducing the frequency of use to once a week or weekends [38]. We did not find significant reductions in measures of opioid use or nonmedical stimulant use frequency. However, there were low numbers of participants who used these substances in the study, particularly nonmedical stimulants. This finding may be related to the way in which the current *Minder* app allows users to select whatever content they think is most relevant to them regardless of their current mental health or substance use status. Substance use is often perceived by students to be higher than it actually is among their peers, thus normalizing its use on university campuses [39,40]. As a result, students may not be as motivated to address substance use compared to other aspects of their lives in which they may be experiencing distress. Previous studies have found that attitudes and norms surrounding drinking predict alcohol use behaviors among college students [41,42]. Addressing existing positive attitudes regarding commonly used substances (eg, alcohol and cannabis), as well as variations in norms such as for different genders [43], may be important in improving engagement with this content and tailoring the app to the needs of users. Future versions of the *Minder* app could include more nuanced approaches to address potentially harmful norms by providing tailored recommendations for substance use content within the app.

These findings are promising when considering the potential benefits of the *Minder* app as a tool in more comprehensive approaches to campus mental health that include early intervention and prevention. Given the extensive mental health needs of university students, stepped-care approaches have been identified as an efficient strategy to organize the delivery of campus mental health services in Canada [44]. The *Minder* intervention, which requires few resources (support is limited to online formats and provided by volunteer student coaches), could be readily integrated into such systems to support self-screening (via the existing services component and completion of the PHQ-9 and GAD-7) and the provision of immediate support and resources for students without higher levels of clinical concerns. It is also important to note that the *Minder* app was co-designed with students to connect users with the broader campus mental health systems through the Services component as well as with the greater student body using the Community component. In this way, it can be used to strengthen the connections between these existing systems and fill gaps in services, particularly in the area of prevention and treatment for mild to moderate symptomology.

### Strengths

A major strength of the *Minder* app is that it has been meaningfully codeveloped with university students and campus health care providers. Many mobile apps are not able to retain users after initial download and have low engagement rates overall [45]. Co-design processes have been identified as an effective means of ensuring that e-tools meet the needs of the end users they are trying to help [15]. The *Minder* app used an extensive codevelopment process that allowed for many improvements to be made with direct input from students and the clinicians currently supporting them [16]. The positive impact and low rates of loss to follow-up in this trial provide some support for the general acceptability of *Minder*. Previous studies on internet-based interventions for university students have also demonstrated their effectiveness in reducing mental health outcomes and that these interventions are generally acceptable to students; however, acceptability is often not reported [9].

Another strength of this study is the pragmatic trial design, which included a large nonclinical sample. Remuneration was only provided to participants for completion of the baseline and follow-up surveys, not for use of the intervention itself. Participants were also not told to use the app in any specific way or for any given amount of time when starting the study. Although this approach may have contributed to the finding of small average effects, it does increase the generalizability of our findings to other populations and provides an estimate of the impact of the app if it were made available to all students.

### Limitations

Several limitations should be considered when interpreting the results of our study. As with many trials for online interventions, the participants were not blinded to what condition they were in, leading to a potential for placebo effects. However, control group participants did download the app and completed the surveys within it, which may have helped mitigate these effects on the trial. There were also several minor technical issues throughout the trial that may have impacted the participants’ experience with the app; however, these were resolved quickly by the research team. An explanation of these issues can be found in Multimedia Appendix 1. There were some participants in the intervention group who did not use the app at all apart from completing the surveys. Given the ITT trial design, all randomized participants were included in the analysis; however, future analyses are planned to examine the effects of the intervention for those who actually engaged with the app content, along with the identification of subgroups that may benefit the most from this type of intervention.

### Implications for Future Research

Further research will be conducted to better understand how to optimize the *Minder* intervention for different needs and additional populations. By better understanding who benefitted from the intervention and what content they used, we plan to make the app more personalized (including recommendations and features). In addition, future codevelopment processes will be needed to further improve app use and incorporation into existing systems of care. There were some participants who did not use the app outside the surveys, so trying to make the intervention more appealing for these users will be important. This may involve gamification strategies, refinement of content and enhancement of the chatbot using artificial intelligence tools, and the development of new features.

### Conclusions

The *Minder* app was effective in reducing symptoms of anxiety and depression, with provisional support for increasing mental well-being and reducing the frequency of cannabis and alcohol use in a general population of university students. These findings support our use of a codevelopment approach and provide evidence of the potential of digital intervention tools such as *Minder* to support prevention and early intervention efforts for university students.

## Appendix

Multimedia Appendix 1 Technical issues and protocol changes.

Multimedia Appendix 2 Chatbot activity content overview.

Multimedia Appendix 3 Identified secondary outcomes with scoring.

Multimedia Appendix 4 CONSORT-eHEALTH checklist (V 1.6.1).

## Abbreviations

AUDIT: Alcohol Use Disorders Identification Test
GAD-7: 7-item General Anxiety Disorder scale
GLMM: generalized linear mixed-effects model
IRR: incidence rate ratio
ITT: intention-to-treat
MAR: missing at random
PHQ-9: 9-item Patient Health Questionnaire
UBC: University of British Columbia
USAUDIT-C: US Alcohol Use Disorders Identification Test–Consumption Scale
