# Supplementary material for: Effectiveness of the Minder Mobile Mental Health and Substance Use Intervention for University Students: Randomized Controlled Trial
Source: J Med Internet Res. 2024 Mar 27;26:e54287. doi: 10.2196/54287 (PMC11007604; doi:10.2196/54287)
Supplement: Multimedia Appendix 1 [file jmir_v26i1e54287_app1.docx]

**Appendix A.** **Technical issues and protocol changes**

**Minor Protocol Changes.**

| Change Type | Description | Date of change |
| --- | --- | --- |
| Logistical change | The eligibility screener question was clarified following a suggestion from a participant. The previous screener for suicide plan was worded as follows “In order to make sure that this app is appropriate for you at this time, we need to ask a question about your personal safety. Do you have a current suicidal plan?”. | October 2022 |
| Logistical change | An additional check was added to validate that students could only register for the study once with their student ID. | October 2022 |
| Logistical change | Addition of a second reminder email to complete the baseline survey and a third reminder to complete the follow up survey. | November 2022 |
| Technical change | Notification for when a peer coach was assigned to a user added. | February 2023 |
| Logistical change | Addition of a third reminder email to complete the baseline survey several months after originally enrolling in the study. | April 2023 |

**Technical issues.**

| Change Type | Description |
| --- | --- |
| Technical issue | Peer coaching outage—participants were not able to access their coaches for one week during a system issue. Impacted participants were contacted once the issue was resolved. |
| Technical issue | Several automated reminder emails to complete surveys were sent in error due to a system glitch. An error was sent to notify participants about the error. |
| Technical issue | Several images within the app experienced a technical issue that resulted in them not loading. This issue was resolved. |
| Technical issue | The automated randomization function experienced a glitch which resulted in around 10 participants being assigned according to the previous block. |
| Logistical deviation | Two participants in the intervention were assigned to a coach late due to a human error. |
