# Supplementary material for: Effectiveness of the Minder Mobile Mental Health and Substance Use Intervention for University Students: Randomized Controlled Trial
Source: J Med Internet Res. 2024 Mar 27;26:e54287. doi: 10.2196/54287 (PMC11007604; doi:10.2196/54287)
Supplement: Multimedia Appendix 2 [file jmir_v26i1e54287_app2.docx]

**Appendix B. Chatbot Activity Content Overview.** *Adapted from Vereschagin et al. (2023)*

| **Category** | **Topic** | **Description** | **Summary Available** |
| --- | --- | --- | --- |
| University Life | Improving Time Management | Provides basic tips based on CBT for better managing time, especially in relation to school. | Yes |
|  | Overcoming Failure | A student talks about dealing with failures at university. This is followed by an activity to help identify your own strengths to build self-esteem. | Yes |
|  | Adapting to University Life | Provides tips for new students from other students about transitioning to university, graduate school, or between programs. | No |
|  | Managing Impulsive Reactions | Explains and provides strategies to deal with impulsivity based on CBT. | Yes |
|  | Hear from the UBC Community | Video series from members of the UBC community which explain their own experiences supporting students with mental health and substance use and how to access help. | No |
|  | A Welcome from Santa Ono | Introduction to the app by the president of the university. | No |
|  | Setting Substance Use Boundaries | Teaches skills on assertive communication and how to refuse substances in social situations. | Yes |
| Wellness | Solving Problems | Outlines a strategy to work through problems using CBT skills. | Yes |
|  | Exploring Self-Care | Identifies self-care strategies that are already used by the participant and provides ideas for other ways to practice self-care across different areas of their life. | Yes |
|  | Improving My Sleep | Provides a variety of strategies to get better sleep based on CBT. | Yes |
|  | Facing Mental Health Stigma | Provides psychoeducation on the different areas of stigma (self, societal, and others) and identifies ways to deal with each of these. | Yes |
|  | Practicing Mindfulness | A series of 360-degree videos filmed in different nature settings that walk through guided mindfulness activities. | No |
|  | Hear from the UBC Community | Video series from members of the UBC community which explain their own experiences supporting students with mental health and substance use and how to access help. | No |
| Relationships | Managing Impulsive Reactions | Explains and provides strategies to deal with impulsivity based on CBT. | Yes |
|  | Dealing with Loneliness | Explores feelings of loneliness and suggests ways to connect better with others through behavioral activation and CBT. | Yes |
|  | Navigating Relationships | Explains skills to build and maintain relationships with other people, as well as communicate in an assertive manner based on DBT. | Yes |
| Sadness | Exploring Thinking Styles | Explains the different types of attributional styles and how to avoid making one-sided appraisals by instead considering more balanced explanations of a situation. Content is based on MCT. | Yes |
|  | Dealing with Sadness | Explains how feelings, triggering situations, automatic thoughts, and maladaptive behaviors are related based on CBT. | Yes |
|  | Learning about Thinking Traps | Reviews the different types of cognitive distortions and allows them to practice identifying these distortions in their own thoughts. Content is based on CBT. | Yes |
|  | Challenging Negative Thoughts | Explains how to challenge automatic thoughts and reformulate them in a more realistic way. Content is based on CBT. | Yes |
|  | Coping with Overwhelming Thoughts | Explains what overwhelming and ruminative thoughts are and provides strategies to cope with them once they are identified, including mindfulness and grounding techniques. Content is based on MCT. | Yes |
| Stress & Anxiety | Challenging Stress & Anxiety | Explains how feelings, triggering situations, automatic thoughts, and maladaptive behaviors are related. Content is based on CBT. | Yes |
|  | Learning about Thinking Traps | Reviews the different types of cognitive distortions and allows them to practice identifying these distortions in their own thoughts. Content is based on CBT. | Yes |
|  | Challenging Unhelpful Thoughts | Explains how to challenge automatic thoughts and reformulate them in a more realistic way. Content is based on CBT. | Yes |
|  | Coping with Overwhelming Thoughts | Explains what overwhelming and ruminative thoughts are and provides strategies to cope with them once they are identified, including mindfulness and grounding techniques. Content is based on MCT. | Yes |
|  | Grounding Exercise | A brief video to help manage feelings of anxiety in the moment by being mindful of your surroundings (i.e., things you can see, hear, and feel). | Yes |
|  | Overcoming Fear | Provides strategies to identify fears and take small steps to gradually challenge and overcome them. Content is based on CBT. | Yes |
|  | Practicing Mindfulness | A series of 360-degree videos filmed in different nature settings that walk through guided mindfulness activities. | No |
|  | Exploring Thinking Styles | Explains the different types of attributional styles and how to avoid making one-sided appraisals by instead considering more balanced explanations of a situation. Content is based on MCT. | Yes |
| Substance Use | Assessing My Drinking | Provides personalized and normative feedback on their drinking behavior compared to other peers at the university, and delivers psychoeducation and strategies to be safer while drinking. | Yes |
|  | Tracking My Drinks | A tool to keep track of drinking and goals in real-time. | No |
|  | Reflecting On My Alcohol Use | Explores feelings and consequences related to the individual’s current alcohol use and any pros and cons of reducing use. Also, allows the individual to set goals based on their current readiness to change. Content is based on CBT and Motivational Interviewing. | No |
|  | Assessing My Cannabis Use | Provides personalized and normative feedback on cannabis use compared to other peers at the university, and delivers psychoeducation related to the lower-risk cannabis guidelines. | Yes |
|  | Reflecting On My Cannabis Use | Explores feelings and consequences related to the individual’s current cannabis use and any pros and cons of reducing use. Also, allows the individual to set goals based on their current readiness to change. Content is based on CBT and Motivational Interviewing. | No |
|  | Practicing Mindfulness | A series of 360-degree videos filmed in different nature settings that walk through guided mindfulness activities. | Yes |
|  | Improving My Sleep | Provides a variety of strategies to get better sleep. Content is based on CBT. | Yes |
|  | Grounding Exercise | A brief video to help manage feelings of anxiety in the moment by being mindful of your surroundings (i.e., things you can see, hear, and feel). | Yes |
|  | Reflecting On My Stimulant Use | Discusses the individual’s motivations for using stimulants with a focus on seeking external support. Content is based on CBT and Motivational Interviewing. | No |
|  | Learning About Stimulants | Provides normative feedback on stimulants, and delivers psychoeducation focusing on risks associated with both street and prescription stimulants. | Yes |
|  | Improving Time Management | Provides basic tips for better managing time, especially in relation to school. Content is based on CBT. | Yes |
|  | Reflecting On My Opioid Use | Discusses motivations to use opioids with a focus on seeking external support. Content is based on CBT and Motivational Interviewing. | No |
|  | Learning About Opioids | Provides general psychoeducation focused on the risks of using opioids and how to respond to an overdose. | Yes |
|  | Setting Substance Use Boundaries | Teaches skills on assertive communication, body language and how to refuse substances in social situations. | Yes |

CBT= Cognitive Behavioural Therapy, MCT= Metacognitive Training, DBT= Dialectical Behavioural Therapy
