# Supplementary material for: Effectiveness of the Minder Mobile Mental Health and Substance Use Intervention for University Students: Randomized Controlled Trial
Source: J Med Internet Res. 2024 Mar 27;26:e54287. doi: 10.2196/54287 (PMC11007604; doi:10.2196/54287)
Supplement: Multimedia Appendix 3 [file jmir_v26i1e54287_app3.docx]

**Appendix C. Identified secondary outcomes with scoring**

In the past 30 days, how often did you use cannabis?

- - (0) Not in the past 30 days
  - (1) 1 day in the past 30 days
  - (2) 2 or 3 days in the past 30 days
  - (3) 1 or 2 day(s) per week
  - (4) 3 or 4 days per week
  - (5) 5 or 6 days per week
  - (6) Every day: 1 or 2 times a day
  - (7) Every day: 3 or more times a day
  - I don’t know
  - I prefer not to answer

*Thinking back over the past 30 days:*

- - How often do you have a drink containing alcohol?
    - (0) Never
    - (1) Less than monthly
    - (2) Monthly
    - (3) Weekly
    - (4) 2-3 times a week
    - (5) 4-6 times a week
    - (6) Daily
  - How many drinks containing alcohol do you have on a typical day when you are drinking?
    - (0) 1 drink
    - (1) 2 drinks
    - (2) 3 drinks
    - (3) 4 drinks
    - (4) 5-6 drinks
    - (5) 7-9 drinks
    - (6) 10 or more drinks
- How often do you have 5 (if your sex assigned at birth was male) OR 4 (if your sex assigned at birth was female) or more drinks on one occasion?
  - - (0) Never
    - (1) Less than monthly
    - (2) Monthly
    - (3) Weekly
    - (4) 2-3 times a week
    - (5) 4-6 times a week
    - (6) Daily

Have you used any of the following opioids in the past 30 days? Please select all that apply:

- - Pharmaceutical opioid taken as prescribed by a healthcare professional
  - Pharmaceutical opioid bought over-the-counter and taken as recommended
  - Pharmaceutical opioid taken without a prescription or in larger doses than prescribed/recommended to get high, buzzed, numbed out, or for any other reason
  - Any street opioid
  - I have not used any of the above in the past 30 days

For each selected response:

- - - In the past 30 days, how often did you use any xyz (replace with selected response):
      - Never (0)
      - 1-3 days a month (1)
      - 1-2 days a week (2)
      - 3-4 days a week (3)
      - Every or nearly every day (4)

Have you used any of the following stimulants in the past 30 days? Please select all that apply:

- Pharmaceutical stimulant taken as prescribed by a healthcare professional
- Pharmaceutical stimulant taken without a prescription or in larger doses than prescribed to get high, buzzed, numbed out, to help you study, or for any other reason
- Any street stimulant
- I have not used any of the above in the past 30 days

For each selected response:

- - - In the past 30 days, how often did you use any xyz (replace with selected response):
      - Never (0)
      - 1-3 days a month (1)
      - 1-2 days a week (2)
      - 3-4 days a week (3)
      - Every or nearly every day (4)
